# Western Texas Medical Association

**Published:** 1900-01

**Authors:** 


### Western Texas Medical Association.—Meeting December 28, 1899.—Important Resolutions Adopted.

Members present: Drs. S. Burg, B. F. Kingsley, John S. Lankford, W. E. Luter, Frank Paschal, A. S. McDaniel, C. E. R. King, Alfred C. McDaniel, J. H. Bindley, H. A. Tutwiler, P. Baldesarelli, and J. W. Morrow.

Minutes of the previous meeting read, and, upon motion, approved.

Under the head of new business, Dr. Frank Paschal offered the following motion, which was carried, namely, that a committee of seven be appointed with the President, Dr. Burg, as chairman, to communicate with our members of Congress and State medical societies to induce them to unite in an effort to get Congress to establish in the different parts of the Southwest institutions for the care of indigent consumptives throughout the United States.

The committee appointed is as follows: S. Burg, chairman; F. Paschal, B. F. Kingsley, C. E. R. King, H. A. Tutwiler and John V. Spring.

Following this a resolution was offered by Dr. B. F. Kingsley, which was as follows.

"Inasmuch as a bill has been introduced into the United States Senate for the third time by Senator Gallinger, of New Hampshire, for the further prevention of cruelty to animals in the District of Columbia, and known as Senate bill No. 34, the object of which, according to its advocates, is twofold: First, to prohibit vivisection; and, second, to aid the passage of similar bills in all the State Legislatures.

"*Resolved,* That it is our duty as a body of scientific men to do all in our power to defeat this insidious and inhuman effort to restrain scientific research, to stop the progress of preventive medicine, and to perpetuate ignorance and disease and suffering among animals and men.

"*Resolved,* That this Association exert its utmost influence, individually and collectively, with the Senators and Representatives in Congress from this State, and with the members of the Senate committee in charge of the bill, to defeat the same, and that a copy of these resolutions be forwarded to each one of these gentlemen respectively.

"*Resolved,* That a petition be circulated among the citizens of San Antonio in order to arouse as vigorous a protest to this infamous and misguided scheme as possible."

The resolution elicited a lively discussion, and, upon motion by Dr. Kingsley, seconded by Dr. Paschal, was adopted; Dr. C. E. R. King opposing its adoption.

The following committee was, upon motion, appointed to carry out the ideas expressed in the resolutions: Drs. Kingsley, Burg, Paschal, Tutwiler and Lankford.

Upon motion made by Dr. Tutwiler, a committee of three was appointed to draw up the following resolution:

*Resolved,* That this Association call the attention of the Governor to the necessity of better treatment of our insane, both by appointing consulting gynecologists to each lunatic asylum, and by increasing the salaries of the superintendents to not less than $3500 per annum and maintenance for his family, and that the appointments be taken from politics as much as possible.

Adopted. Committee: Drs. Tutwiler, Luter and Kingsley.

The report of Dr. Lankford, who was appointed a committee of one to investigate the relationship existing between our local and State Associations, was to the effect that he was in favor of paying our dues and keeping in affiliation with the State Association. His report was accepted.

Dr. C. E. R. King was appointed in place of Dr. B. E. Hadra (removed) on the committee to investigate the uniting of the Austin District Society with the Western Texas Medical Association, and Dr. Kingsley was made its chairman.

Several interesting genito-urinary cases were presented for discussion. Dr. Paschal, who has had considerable experience with this class of work, reported a case upon which he recently operated for stone in the bladder, doing the combined operation of internal and external urethrotomy; he believes that the combined operation is calculated to give the most happy results.

